# Ethnomedical Survey of the Plants Used by Traditional Healers in Narok County, Kenya

**DOI:** 10.1155/2019/8976937

**Published:** 2019-01-01

**Authors:** Gabriel Kigen, Zipporah Kamuren, Evangeline Njiru, Bernard Wanjohi, Wilson Kipkore

**Affiliations:** ^1^Department of Pharmacology and Toxicology, Moi University School of Medicine, P.O. Box 4606-30100, Eldoret, Kenya; ^2^Department of Internal Medicine, Moi University School of Medicine, P.O. Box 4606-30100, Eldoret, Kenya; ^3^Department of Wildlife Management, University of Eldoret, P.O. Box 1125-30100, Eldoret, Kenya; ^4^Department of Forestry, University of Eldoret, P.O. Box 1125-30100, Eldoret, Kenya

## Abstract

Most of the plants used by herbalists amongst the various Kenyan communities have not been documented despite their widespread use. The purpose of this research was to document the medicinal plants used by the herbalists from the Maasai, a community that still relies on herbal medicine to a large extent for the provision of medical services. Semistructured interviews, direct observations, group discussions, and in-depth interviews were used to collect information from the traditional healers. A total of 47 plant species belonging to 31 families were identified. They were used in the treatment of 33 medical and 4 veterinary conditions.

## 1. Introduction

Medicinal plants still play an important role in primary healthcare in many sub-Saharan African countries due to variety of reasons including lack of health services, cultural norms, and traditional beliefs [1–3]. Many patients in these countries combine traditional medicine (TM) with conventional medicine, especially those with chronic diseases [4]. The use of TM is in most cases widespread and not only limited to the rural areas or low-income settings, but also in urban and well to do settings [3, 5, 6]. In addition, there is a growing global demand for traditional and complementary medicine [2]. The knowledge of these medicinal preparations is therefore important in order to enable health practitioners to be aware of the kind of TM their patients are on, so as to minimize potential adverse effects resulting from herb-drug interactions [7]. The documentation of the type of medicinal plants used by the traditional medical practitioners (TMPs) and the conditions treated is crucial to this endeavor [4]. In addition, databases containing this information would also be important for research and potential development of new drugs, as many of the drugs in current use have been developed from medicinal plants [8, 9]. Examples of these include paclitaxel, an antitumour drug developed from the bark of* T. brevifolia* [10], the antimalarial drug artemisinin from* Artemisia annua* [11], digoxin from* Digitalis lanata*, atropine from* Atropa belladona, *aspirin from* Filipendula ulmaria, *and several other drugs. However, despite the widespread use of medicinal plants in Kenya, several have not been documented [4, 12]. The main aim of this research was to document the medicinal plants used by the Maasai community in Narok County, one of the regions in Kenya whereby the practice of herbal medicine is still widespread for future research.

## 2. Materials and Methods

### 2.1. Study Area

**Figure 1:**
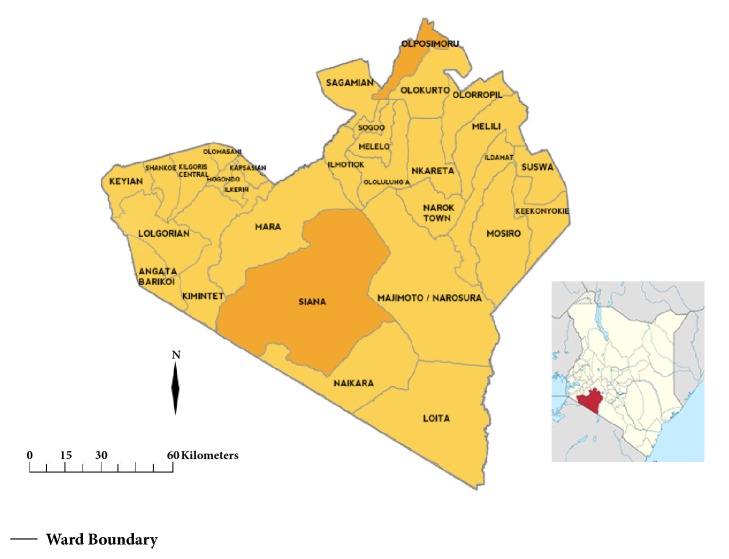
Map of Narok County showing Olpusimoru and Siana wards and its position within Kenyan map.

The study was conducted in two locations within Narok County, Olpusimoru (2°1′0′′S, 36°54′0′′E) a highland area located in the Northern part; and Sekenani (1°30′58.33′′S, 35°20′19.63′′E) a lowland area located in the South-Western region [Siana Ward] [Figure 1]. Olpusimoru is a mountainous forested terrain with an average altitude of 2478 metres and high rainfall, while Sekenani has an average altitude of 1820 m with comparatively low rainfall. It borders the globally famous Maasai Mara National Reserve on the East [13, 14].

### 2.2. Data Collection

Ethnobotanical data was collected from TMPs between March and December 2016. The research team is comprised of professionals from the medical field and botany, including a physician (EN), two clinical pharmacologists (GK and ZK), a taxonomist (BW), and a plant specialist (WK). There were also two local lead persons, one from each site who accompanied the team at each visit in order to direct and introduce them to the TMPs. All TMPs that we met were willing to participate in the research. A total of 37 TMPs comprising 20 men and 17 women aged between 42 and 85 were interviewed. Semistructured interviews, direct observations, group discussions, and in-depth interviews were used to collect ethnopharmacological information [15–17]. The participant's biodata, conditions they treated, methods of treatment, medicinal plants used, methods of preparation and administration, and dosing forms were recorded. They were also asked to explain the manner in which they arrived at a diagnosis. At the end of each interview, the informants were requested to accompany the research team to sites where they collected the plants and assist in identification. Preliminary identification of the plants was then done by BW and WK, and the plants and their surrounding habitats photographed. The voucher specimens were then collected using standard botanical procedures, and further identification and confirmation were performed using the relevant taxonomic keys at University of Eldoret Herbarium where the specimens were subsequently deposited [18, 19]. The data was then compared to related research that has been carried out in the region.

### 2.3. Data Analysis

The medicinal importance of each plant species used was calculated as per the use-value index for each plant species* (UVs)* using the formula:

(1) $$\begin{array}{c} UVs=\frac{U}{N} \end{array}$$

where *U* is the number of different uses mentioned by each TMP (informant) whereas N is the total number of TMPs interviewed during the survey [20, 21]. The* UV* index theoretically varies from 0, which implies that none of the informants mention any use of the plant, to 1 whereby the plant is most frequently mentioned as useful in treatment of the highest number of conditions.

## 3. Results and Discussion

A total of 47 plant species belonging to 31 families were identified, out of which 36 (77%) were from the lowland area (Sekenani), while 11 (23%) species were from the highlands (Olpusimoru) [Table 1]. The plant details including the voucher numbers are outlined in Table 2.

*Medicinal Plant Uses*. The plants were used in the treatment of 33 medical and 4 veterinary disorders. The detailed list of the plants and their respective medicinal uses are outlined in Table 2 and Supplementary Material section (available here). The most frequently used plant was* Solanum incanum *which had seven medical uses (*UV* = 0.19) followed by* Olea europaea subsp. cuspidata *which had five applications (*UV* = 0.14).* Asparagus africanus*,* Carissa edulis, Commiphora africana, Elaeodendron buchananii, *and* Kigelia africana* had four medicinal uses each (*UV* = 0.11).

Most of the plants used by traditional healers in Kenya have not documented despite the imminent risk of disappearance of this plants due to several factors including deforestation and overexploitation [4]. In addition, the practice is usually a guarded family secret, and some of the siblings may not be willing to inherit the art due to changing lifestyles [22]. The lack of adequate regulation of the practice in Kenya has also led to infiltration by several quacks. However, the Maasai is one community in Kenya which still practices TM to a large extent owing to several reasons including lack of adequate health facilities and traditional values [23]. Some of the reported plants have been evaluated* in vitro* and found to exhibit pharmacological activities related to the uses described by the TMPs [24]. These include* Aerva javanica, Asparagus africanus, Carissa edulis, Sida cuneifolia, and Solanum incanum *which have demonstrated to possess antibiotic/antifungal activities [25–28], while* Gloriosa superba* that is used as an abortifacient has oxytocic activity [29]. The plants used by the TMPs are largely similar to those used by their Kalenjin counterparts that we have reported before, although for different medicinal uses [3, 12, 30, 31]. Additionally, the methods of preparations are slightly different as the Maasai TMPs tend to use a lot of cold herbal infusions prepared by soaking the plant parts in water and hardly use the burnt leaves/barks as their counterparts

## 4. Conclusions

It is important to document traditional medicinal plants used by the various communities in Kenya in order to develop a database for future research. The risk of the rapid disappearance of the knowledge on traditional medicine calls for an urgent multidisciplinary approach towards conserving the information before it is lost forever. Some of these plants may contain undiscovered pharmacological properties which can serve as ingredients for the development of new drugs as has happened in Asia with the discovery of artemisinin. Additionally, medical personnel would also have an idea of the kind of herbal medicine that their patients may be taking and therefore minimize toxic effects through herb-drug interactions.

## Figures and Tables

**Table 1:** Classification of medicinal plants.

	**Family**	**Species**	
		No	*Members*

1.	FABACEAE	5	*Albizia gummifera, Dichrostachys cinerea, Erythrina senegalensis, Senegalia senegal, Vachellia nilotica*

2.	SOLANACEAE	4	*Physalis peruviana, Solanum arundo, Solanum incanum, Solanum mauense*

3.	AMARANTHACEAE	2	*Achyranthes aspera, Aerva javanica*

4.	APOCYNACEAE	2	*Acokanthera schimperi, Carissa edulis*

5.	BURSERACEAE	2	*Commiphora africana, Ficus sycomorus*

6.	CELASTRACEAE	2	*Elaeodendron buchananii, Mystroxylon aethiopicum*

7.	EUPHORBIACEAE	2	*Clutia abyssinica, Croton dichogamous*

8.	MALVACEAE	2	*Grewia bicolor, Sida cuneifolia*

9.	RHAMNACEAE	2	*Rhamnus prinoides, Ziziphus mucronata*

10.	RUBIACEAE	2	*Galium aparinoides, Pavetta subcana*

11.	RUTACEAE	2	*Teclea nobilis, Toddalia asiatica*

12.	APIACEAE	1	*Anthriscus sylvestris*

13.	ASPARAGACEAE	1	*Asparagus africanus*

14.	ASTERACEAE	1	*Acmella calirhiza*

15.	BIGNONIACEAE	1	*Kigelia africana*

16.	BORAGINACEAE	1	*Cordia monoica*

17.	CANELACEAE	1	*Warburgia ugandensis*

18.	CAPPARIDACEAE	1	*Boscia angustifolia*

19.	COLCHICACEAE	1	*Gloriosa superba*

20.	COMMELINACEAE	1	*Aneilema equinoctiale*

21.	CRASSULACEAE	1	*Kalanchoe crenata*

22.	CUCURBITACEAE	1	*Momordica friesiorum*

23.	EBENACEAE	1	*Diospyros abyssinica*

24.	FLACOURTIACEAE	1	*Dovyalis abyssinica*

25.	LABIATAE	1	*Leonotis mollissima*

26.	OLEACEAE	1	*Olea europaea subsp. cuspidata*

27.	PRIMULACEAE	1	*MyrsineaAfricana*

28.	ROSACEAE	1	*Prunus africana*

29.	SANTALACEAE	1	*Osyris lanceolata*

30.	SAPINDACEAE	1	*Pappea capensis*

31.	VITACEAE	1	*Cissus fischeri*

	**Total**	**47**	

**Table 2:** Medicinal plant uses.

**No**	**Botanical Name**	**Family**	**Voucher No.**	**Maa name**	**Habitat**	**Parts used**	**Method of preparation**	**Medicinal uses**
1.	*Achyranthes aspera *L.	AMARANTHACEAE	OLP/08/15/007	*Olerubat*	Highland	*Roots*	*Boiled*	*Arthritis*

2.	*Acmella calirhiza *Del	ASTERACEAE	OLP/08/15/009	*Ekum*	Highland	*Flowers*	*Crushed and mixed with water*	*Oral thrush in children*

3.	*Acokanthera schimperi* (A.DC.) Schweinf	APOCYNACEAE	MAU/08/15/027	*Olmorijioi*	Lowland	*Roots*	*Boiled*	*Syphilis*
						*Bark*	*Soaked in water*	*Arrow poison*

4.	*Aerva javanica * (Burm.f.) Shult.	AMARANTHACEAE	MAU/08/15/032	*Eleleshwa-ekop*	Lowland	*Flowers*	*Ground into paste & mixed with water*	*East Coast Fever in cattle*

5.	*Albizia gummifera *(J.F. Gmel.) C.A.Sm.	FABACEAE	MAU/08/15/018	*Osupakupe*	Lowland	*Pods*	*Crushed*	*Stomachache*
						*Roots*	*Pounded*	*Skin disorders*

6.	*Aneilema aequinoctiale* P. Beauv	COMMELINACEAE	MAU/08/15/020	*Enkaiteteyiai*	Lowland	*Leaves*	*Soaked in water*	*Malnutrition, colds*
						*Flowers*	*Pressed to produce juice*	*Ocular disorders*

7.	*Anthriscus sylvestris* (L.) Hoffm	APIACEAE	MAU/08/15/028	*Oldule*	Lowland	*Seeds*	*Mixed with honey and chewed*	*Chesty colds*

8.	*Asparagus africanus* Lam	ASPARAGACEAE	MAU/08/15/006	*Empereempapa*	Lowland	*Leaves, stem & roots*	*Soaked in water*	*Mental illness*
						*Leaves*	*Soaked in water*	*Wounds*
						*Roots*	*Soaked in water*	*Venereal diseases*
							*Chewed*	*Cough & sore throat*

9.	*Boscia angustifolia *Harvey	CAPPARIDACEAE	MAU/08/15/001	*Oloireroi*	Lowland	*Leaves*	*Pounded*	*Cattle fever*
						*Bark*	*Crushed & mixed with water*	*Gynaecological disorders*

10.	*Carissa edulis *Harv	APOCYNACEAE	MAU/08/15/003	*Olamuriaki*	Lowland	*Roots*	*Boiled*	*Lower abdominal pains in pregnancy, gonorrhea, chest pains, polio symptoms*

11.	*Cissus fischeri *Gilg	VITACEAE	MAU/08/15/011	*Osurkurtuti*	Lowland	*Leaves*	*Soaked in water*	*Respiratory disorders in cattle*

12.	*Clutia abyssinica *Jaub. & Spach.	EUPHORBIACEAE	OLP/08/15/011	*Enkiparnyeny*	Highland	*Roots*	*Boiled*	*Appetizer*

13.	*Commiphora africana *(A. Rich.) Endl	BURSERACEAE	MAU/08/15/034	*Osilalei*	Lowland	*Roots*	*Boiled*	*Swollen testicles, abdominal pains*
						*Bark*	*Chewed*	*Snake bite*
						*Fruits*	*Boiled*	*Typhoid*

14.	*Cordia monoica* Roxb	BORAGINACEAE	MAU/08/15/031	*Oseki*	Lowland	*Leaves, bark*	*Leaves- boiled, bark - pounded*	*Leprosy*
						*Roots*	*Boiled*	*Mental illness*
						*Leaves*	*Pounded*	*Ocular disorders*

15.	*Croton dichogamous Pax*	EUPHORBIACEAE	MAU/08/15/008	*Ollokirdangai*	Lowland	*Roots*	*Boiled*	*Polio-like symptoms, gonorrhea, chest pain*

16.	*Dichrostachys cinerea* Wight et Arn	FABACEAE	MAU/08/15/009	*Emerrumori*	Lowland	*Leaves*	*Pounded*	*Local anaesthesia, ulcers, gonorrhea*

17.	*Diospyros abyssinica* Hiern	EBENACEAE	MAU/08/15/025	*Olchartuyian*	Lowland	*Bark*	*Pounded & soaked in water*	*Malaria, ocular d/orders in livestock*

18.	*Dovyalis abyssinica* (A. Rich.) Warb	FLACOURTIACEAE	OLP/08/15/006	*Olmorogi*	Highland	*Roots*	*Boiled*	*Gonorrhea*
						*Leaves*	*Chewed*	*Toothache*

19.	*Elaeodendron buchananii *(Loes) Loes.	CELASTRACEAE	MAU/08/15/004	*Osoket*	Lowland	*Roots*	*Dried and ground to powder*	*Wounds, syphilis*
						*Roots*	*Boiled or dried and ground to powder*	*Respiratory d/orders*
						*Leaves*	*Chewed*	*Diarrhoea*

20.	*Erythrina senegalensis DC.*	FABACEAE	MAU/08/15/036	*Ol-oboni*	Lowland	*Roots*	*Boiled*	*Polio-like symptoms, gonorrhea, chest pain*

21.	*Ficus sycomorus *L.	BURSERACEAE	MAU/08/15/024	*Olngaboli*	Lowland	*Roots*	*Boiled, chewed*	*Abortifacient*

22.	*Galium aparinoides *Forssk	RUBIACEAE	MAU/08/15/026	*Olngeriantus*	Lowland	*Whole plant*	*Pounded & soaked in water or boiled*	*Throat cancer in cattle*

23.	*Gloriosa superba *L.	COLCHICACEAE	MAU/08/15/022	*Sakutayei*	Lowland	*Roots*	*Chewed or soaked in water*	*Abortifacient*

24.	*Grewia bicolor*	MALVACEAE	MAU/08/15/014	*Ositeti*	Lowland	*Roots*	*Soaked in water*	*Respiratory d/orders, snake bite*

25.	*Kalanchoe crenata *(Andrews) Haw	CRASSULACEAE	OLP/08/15/008	*Ormasilig*	Highland	*Leaves*	*Warmed*	*Poultice*

26.	*Kigelia africana * (Lam.) Benth.	BIGNONIACEAE	MAU/08/15/021	*Oldarpoi*	Lowland	*Fruits*	*Brewed*	*Measles in children*
						*Roots*	*Boiled*	*Abortifacient*
						*Bark*	*Boiled*	*Headache*
						*Leaves*	*Boiled*	*Malaria*

27.	*Leonotis mollissima* Guerke	LABIATAE	OLP/08/15/003	*Olbibi*	Highland	*Leaves*	*Soaked in water or boiled*	*Antiseptic, skin rashes, blood purifier*

28.	*Momordica friesiorum* (Harms) C. Jeffrey	CUCURBITACEAE	OLP/08/15/001	*Esumeito*	Highland	*Roots*	*Pounded & mixed with water*	*Induce vomiting*

29.	*Myrsine Africana *L.	PRIMULACEAE	OLP/08/15/004	*Seketet*	Highland	Seeds	*Ground*	*Antihelminthic, heartburn*

30.	*Mystroxylon aethiopicum *(Thunb.) Loes.	CELASTRACEAE	MAU/08/15/035	*Olodonganayioi*	Lowland	*Bark*	*Boiled*	*Colic pain, especially in children*

31.	*Olea europaea subsp. cuspidata* (Wall. ex G. Don) Cif.	OLEACEAE	MAU/08/15/023	*Oloirien*	Lowland	*Bark*	*Pounded and soaked in water*	*Antihelminthic*
						*Leaves*	*Boiled*	*Liver disease*
						*Roots*	*Boiled*	*Polio-like symptoms, gonorrhea, chest pain*

32.	*Osyris lanceolata *Hochst. & Steud. ex A. DC.	SANTALACEAE	MAU/08/15/016	*Olosesiai*	Lowland	*Bark*	*Boiled*	*Abdominal pains in children*
						*Leaves*	*Pounded*	*Diarrhoea*
						*Roots*	*Boiled*	*Gonorrhea*

33.	*Pappea capensis* Eckl. & Zeyh	SAPINDACEAE	MAU/08/15/029	*Olkisik-ongo*	Lowland	*Bark*	*Boiled*	*Abdominal disorders*

34.	*Pavetta subcana *Hiern.	RUBIACEAE	MAU/08/15/002	*Olabai*	Lowland	*Roots*	*Boiled*	*Gonorrhea*
						*Whole plant*	*Soaked in water*	*Cough in calves, fleas*

35.	*Physalis peruviana *L.	SOLANACEAE	OLP/08/15/010	*Ormumai*	Highland	*Roots*	*Squeezed/chewed*	*Tonsillitis*

36.	*Prunus africana * (Hook.f.) Kalkman	ROSACEAE	MAU/08/15/012	*Olkujuk*	Lowland	*Leaves*	*Pounded & soaked in water*	*Appetizer*
						*Bark*	*Pounded & mixed with water*	*Stomachache*

37.	*Rhamnus prinoides *L'Hér.	RHAMNACEAE	OLP/08/15/002	*Olkonyel*	Highland	*Roots*	*Boiled*	*Gonorrhea, arthritis*
						*Stem*	*Pounded and mixed with water*	*Preservative*

38.	*Senegalia senegal * (L.) Britton & P. Wilson	FABACEAE	MAU/08/15/010	*Oitioibor*	Lowland	*Roots*	*Boiled*	*Purgative, constipation & gonorrhea*
						*Bark*	*Boiled*	*Diarrhoea & abdominal disorders*

39.	*Sida cuneifolia *Roxb	MALVACEAE	MAU/08/15/005	*Olonini*	Lowland	*Roots*	*Chewed*	*Sore throat*
							*Boiled*	*Reduce foetal movements in pregnancy*

40.	*Solanum arundo*	SOLANACEAE	MAU/08/15/013	*Esokawai*	Lowland	Roots	*Chewed, pounded & soaked in water*	*Fever*

41.	*Solanum incanum* L.	SOLANACEAE	MAU/08/15/015	*Entulelei*	Lowland	*Roots*	*Boiled*	*Abdominal pains, fever*
							*Raw roots used*	*Toothache*
						*Leaves*	*Chewed and applied*	*Snake bite*
						*Fruits*	*Juice*	*Chest pain, wounds & skin disorders, Respiratory disorders in sheep*

42.	*Solanum mauense* Bitter.	SOLANACEAE	MAU/08/15/007	*Olesayiet*	Lowland	*Berries*	*Cooked*	*Pneumonia*
						*Roots*	*Boiled*	*Anthrax in both humans and animals*

43.	*Teclea nobilis* Del.	RUTACEAE	MAU/08/15/019	*Olgilai*	Lowland	*Leaves, roots*	*Boiled*	*Pneumonia, arthritis*

44.	*Toddalia asiatica *(L.) Lam	RUTACEAE	MAU/08/15/030	*Oleparmunyio*	Lowland	*Bark*	*Boiled or soaked in water*	*Respiratory disorders*
						*Roots*	*Boiled*	*Malaria*

45.	*Vachellia nilotica * (L.) P.J.H.Hurter & Mabb	FABACEAE	MAU/08/15/033	*Olkiloriti*	Lowland	*Bark*	*Pounded and mixed with water*	*Stomachache, indigestion*

46.	*Warburgia ugandensis *Sprague.	CANELACEAE	OLP/08/15/005	*Osokonoi*	Highland	*Bark*	*Pound and mixed with water*	*Malaria, abdominal disorders*
							*Boiled, ground to powder*	*Arthritis*

47.	*Zizyphus mucronata *Willd.	RHAMNACEAE	MAU/08/15/017	*Oloilalei*	Lowland	*Roots*	*Soaked in water*	*Snake bite*
						*Bark*	*Boiled*	*Arthritis, stomachache*

## Data Availability

The authors confirm that the data supporting the findings of this study are available within the article and/or its supplementary materials.

## Supplementary Materials

Supplementary Materials Details of the medicinal plant uses.
