# Supplementary material for: Ethnomedical Survey of the Plants Used by Traditional Healers in Narok County, Kenya
Source: Evid Based Complement Alternat Med. 2019 Jan 1;2019:8976937. doi: 10.1155/2019/8976937 (PMC6332960; doi:10.1155/2019/8976937)
Supplement: Supplementary Materials — Details of the medicinal plant uses. [file 8976937.f1.zip › 8976937.f1.zip/Supplementary Material - Medicinal Plant uses_ECAM_2604405.pdf]

Details of the Medicinal Plant uses

1. Abdominal disorders

A decoction obtained by boiling the roots of *Solanum incanum* and/or *Commiphora africana* is used for the treatment of abdominal/colic pains, fever, stomachache and indigestion. A decoction made from the boiled bark of *Zizyphus mucronata* is also used. An infusion from the pounded bark of *Vachellia nilotica* is used for treatment of stomachache and indigestion. An infusion prepared by pounding the brown middle bark of *Warburgia ugandensis* and soaking in water may also be used. An extract obtained from the crushed pods of *Albizia gummifera* or pounded bark *Prunus africana* mixed with water is used as a remedy for stomachache. A concoction made from the boiled bark of *Pappea capensis* mixed with soup is administered for treatment of abdominal disorders. A decoction made from the boiled bark of *Mystroxylon aethiopicum* is used to treat abdominal/colic pain, especially in children. Decoction from the bark of *Osyris lanceolata* is also administered to children to relieve abdominal pains. Ground seeds of *Myrsine africana* are chewed to relive heartburn.

2. Diarrhoea

The leaves of *Elaeodendron buchananii* are chewed and swallowed in order to treat diarrhoea. An infusion made from the pounded leaves of *Osyris lanceolata* is used to stop diarrhoea. A decoction of the bark of *Senegalia senegal* is used to treat both diarrhoea and abdominal disorders.

3. Typhoid

A decoction made from the boiled fruits of *Commiphora africana* is mixed with milk and administered.

4. Laxative

A decoction made from the boiled roots of *Senegalia senegal* has mild purgative effect and is taken to relieve constipation or stomachache.

5. Emetic

The pounded roots of *Momordica friesiorum* are mixed with water and administered to induce vomiting.

6. Anthelminthic agents

Ground seeds of *Myrsine africana* are used as dewormers. An infusion obtained from the bark of *Olea europaea subsp. cuspidate* is used to get rid of tapeworms. The bark is pounded, soaked in water and left overnight.

7. Ulcers

A preparation made from the pounded leaves of *Dichrostachys cinerea* is used for treatment of ulcers.

8. Anaesthetic

An extract from the crushed leaves of *Dichrostachys cinerea* is applied topically to provide local anesthesia.

9. Respiratory disorders

An infusion made by soaking the leaves of *Aneilema aequinoctiale* in water is used for the treatment of colds. During an outbreak of common colds, the same infusion is used as a face wash to prevent contracting the colds. The seeds of *Anthriscus sylvestris* are mixed with honey and chewed as a remedy for chesty colds. The roots *Asparagus africanus* are chewed to treat sore throat and cough. Similarly, the roots of *Sida cuneifolia* are chewed and the resultant juice swallowed to relieve sore throats. The juice/sap obtained from either squeezed or chewed roots *Physalis peruviana* is used for treatment of tonsillitis.

A decoction made from the boiled roots of *Carissa edulis* mixed with those of *Olea europaea subsp. cuspidate, Croton dichogamous* and *Erythrina senegalensis* is used to treat chest pains. Juice obtained from *Solanum incanum* fruits is also taken to treat chest pains. An infusion made from the roots of *Grewia bicolor* is also administered to treat chest pains, while a cupful mixed with cold water is administered for the treatment of colds. A concoction made from the boiled or soaked in water roots and bark of *Toddalia asiatica* is mixed with tea or milk, is also used for the treatment flu and cough. A concoction made from of the roots of *Elaeodendron buchananii* or dried and powdered roots of the same plant is mixed with milk and administered to a patient with haemoptysis (coughing up blood); while berries of *Solanum mauense* are cooked and eaten as cure for pneumonia. A decoction made from either leaves, or roots of *Teclea nobilis* mixed with honey may also be used in the treatment of pneumonia.

10. Malaria

The yellow middle bark of *Diospyros abyssinica* is crushed and soaked in water until the solution turns yellow and then sieved before administering to the patient. An infusion prepared by pounding the brown middle bark of *Warburgia ugandensis* and soaking in water may also be used. Milk is added to the infusion and administered to a patient with malaria. A concoction made from the boiled roots *Toddalia asiatica* mixed with milk is also used for the treatment of malaria. An alternative treatment involves the administration of a decoction made from the boiled leaves of *Kigelia africana***.**

11. Fever

Pounded roots of *Solanum arundo* are soaked in water and administered or chewed as a remedy for high fever. A decoction obtained by boiling the roots of *Solanum incanum* is also used for the management of fever.

12. Headache

A decoction made from the bark of *Kigelia africana* is administered to relive headache.

13. Toothache

The fresh top apical leaves of *Dovyalis abyssinica* are chewed and placed around the affected tooth. *Solanum incanum* roots are rubbed around the affected tooth.

14. Oral thrush in children

The flowers of *Acmella calirhiza* are crushed and mixed with water or milk and applied on the affected areas in the child's mouth.

15. Venereal diseases

15.1 Gonorrhea

An infusion obtained from mixing the roots of *Asparagus africanus* with water is used to treat venereal diseases, including gonorrhea. An alternative treatment involves the use of a decoction made from the boiled roots of either *Carissa edulis, Croton dichogamous, Dovyalis abyssinica, Erythrina senegalensis*, *Olea europaea subsp. cuspidate* or *Senegalia senegal* is mixed with sheep tallow. A preparation made from the pounded leaves of *Dichrostachys cinerea* or boiled roots of *Rhamnus prinoides* may also be used. Other treatments include decoction made from the boiled roots of *Osyris lanceolata* mixed milk or meat soup, or concoction made from the boiled roots of *Pavetta subcana* mixed with meat soup or sheep tallow.

15.2 Syphilis

An extract made by soaking the roots of *Acokanthera schimperi* in water is administered to a patient suffering from syphilis, but in a small quantity as the plant is poisonous. The roots of *Elaeodendron buchananii* (a plant known to be poisonous to livestock) are dried, ground to powder and applied on wounds on the affected areas.

15.3 Orchitis

A decoction made from the boiled roots of *Commiphora africana* is used to treat a condition presenting with swollen testicles.

16. Gynaecological disorders

The crushed inner bark of *Boscia angustifolia,* mixed with water is used to treat various gynaecological disorders. A decoction made from the boiled roots of *Carissa edulis* mixed with sheep tallow is used to treat lower abdominal pains in pregnancy. A concoction made from the

boiled roots of *Sida cuneifolia* mixed with milk is administered to pregnant mothers experiencing excessive foetal activity in order to stabilize the pregnancy.

17. Abortion

In order to induce an abortion in pregnant women, a decoction made from the boiled roots of *Ficus sycomorus* is mixed with milk and administered. For pregnant girls, four finger sized pieces of the roots are chewed, which results in abortion after a short while. The roots of *Gloriosa superba* may also be chewed to induce abortion. An infusion obtained from the soaked roots of the same plant may also be administered to achieve the same effect. Alternatively, a decoction made from the boiled roots of *Kigelia africana* may be used.

18. Ocular disorders

Juice from *Aneilema aequinoctiale* flowers is applied on the affected eye. An alternative treatment involves the use of a preparation made by pounding the leaves of *Cordia monoica* and soaking in water.

19. Skin disorders

Pounded roots of *Albizia gummifera* are mixed with water for bathing to treat skin disorders. Juice from the fruits of *Solanum incanum* may also be applied on the affected areas. A decoction obtained by either boiling or soaking in water the leaves of *Leonotis mollissima* is used to treat skin rashes.

20. Measles

A child suffering from measles is washed with an alcoholic beverage made from the fruits of *Kigelia africana*, and then taken to bed and covered heavily to sweat out.

21. Wounds

An extract obtained from the leaves of *Asparagus africanus* or *Leonotis mollissima* is used for treating wounds. The leaves are pound and soaked in water. An alternative treatment involves the use of fruit juice of *Solanum incanum.* The fruits are broken and contents applied directly to fresh cuts or wounds. The roots of *Elaeodendron buchananii* may also be used. They are dried, ground to powder and applied on the wounds.

22. Liver disease

A decoction made from the boiled leaves of *Olea europaea subsp. cuspidate* is administered for the treatment of hepatic disorders.

23. Arthritis

A concoction made from the boiled roots of *Rhamnus prinoides* or *Achyranthes aspera* mixed with soup is used to treat patients with joint disorders, especially osteoarthritis and related

disorders. A concoction made from either leaves, or roots of *Teclea nobilis* mixed with honey may also be used. A decoction made from the boiled bark of *Warburgia ugandensis* or *Zizyphus mucronata* is also used. Alternatively, the dried bark *Warburgia ugandensis* is ground to powder and administered.

24. Poultice

Warmed leaves of *Kalanchoe crenata* are used to massage/soothe a painful part of the body.

25. Mental illness

An infusion made from the pounded leaves, stem or roots of *Asparagus africanus* and soaked in water is drunk 2 to 3 times a day for treatment of mental illness. A decoction made from the boiled roots of *Cordia monoica* mixed with little milk is used for the same purpose.

26. Leprosy

The patient is exposed to steaming/boiling water pot containing the leaves of *Cordia monoica* under a blanket for a period of time. He/she is then washed with the same decoction followed by the application/rubbing of a paste made from the pounded bark of the same plant on his body.

27. Polio like symptoms

A decoction made from the roots of *Carissa edulis* mixed with those of *Olea europaea subsp. cuspidate, Croton dichogamous* and *Erythrina senegalensis* is used to treat a condition which the TMPs described as manifesting with polio like symptoms. We suspected the condition to be a neuromuscular system disorder.

28. Anthrax

A decoction made from the roots of *Solanum mauense* is used to treat anthrax in both humans and animals.

29. Malnutrition

An infusion made from the leaves of *Aneilema aequinoctiale* is administered to children as a remedy for malnutrition.

30. Appetizer

A decoction from boiled roots *Clutia abyssinica* is mixed with soup and administered in order to improve appetite. An infusion made from pounded leaves of *Prunus africana* soaked in water may also be used.

31. Blood purifier

A decoction obtained by either boiling or soaking in water the leaves of *Leonotis mollissima* is used to cleanse the blood.

32. Snake bites

An extract obtained by chewing the bark of *Commiphora africana* combined with tobacco is applied on the bitten area. An alternative treatment involves the chewing of the tender leaves of *Solanum incanum* and rubbing the extract including the leaves hard into the bitten area. An infusion made by soaking the roots of *Grewia bicolor* or *Zizyphus mucronata* in water is also administered for snake bite treatment.

33. Miscellaneous uses

33.1 Preservative

A decoction made form pounded stem of *Rhamnus prinoides* mixed with water is used as a preservative.

33.2 Arrow poison

An infusion from the bark of *Acokanthera schimperi* soaked in water is used as an arrow poison.

34. Veterinary uses

34.1 East Coast Fever

The flowers of *Aerva javanica* are ground into paste, mixed with water and administered to cattle to treat East Coast Fever. An alternative treatment involves use of *Boscia angustifolia* leaves which are pounded and soaked in water.

34.2 Throat cancer in cattle

The whole of *Galium aparinoides* herb is pounded and soaked in water or boiled and decoction administered.

34.3 Respiratory disorders

An extract obtained by soaking the leaves and stem of *Cissus fischeri* is administered to cattle with respiratory disorders, whereas juice from *Solanum incanum* fruits is squeezed into the sheep's nostrils to treat cough. An infusion made by soaking the whole of *Pavetta subcana* plant is administered to calves to relieve cough. It is also used to wash them to get rid of fleas.

34.4 Ocular disorders

The yellow middle bark of *Diospyros abyssinica* is crushed and soaked in water until the solution turns yellow and then sieved. The drops are then applied onto the affected eye.
