# Supplementary material for: Ethnomedical Survey of the Plants Used by Traditional Healers in Narok County, Kenya
Source: Evid Based Complement Alternat Med. 2019 Jan 1;2019:8976937. doi: 10.1155/2019/8976937 (PMC6332960; doi:10.1155/2019/8976937)
Supplement: Supplementary Materials — Details of the medicinal plant uses. [file 8976937.f1.zip › 8976937.f1.zip/Supplementary Material - Medicinal Plant uses_ECAM_2604406.docx]

**Details of the Medicinal Plant uses**

1. **Abdominal disorders**

A decoction obtained by boiling the roots of *Solanum incanum* and/or *Commiphora africana* is used for the treatment of abdominal/colic pains, fever, stomachache and indigestion. A decoction made from the boiled bark of *Zizyphus mucronata* is also used. An infusion from the pounded bark of *Vachellia nilotica* is used for treatment of stomachache and indigestion. An infusion prepared by pounding the brown middle bark of *Warburgia ugandensis* and soaking in water may also be used. An extract obtained from the crushed pods of *Albizia gummifera* or pounded bark *Prunus africana* mixed with water is used as a remedy for stomachache. A concoction made from the boiled bark of *Pappea capensis* mixed with soup is administered for treatment of abdominal disorders. A decoction made from the boiled bark of *Mystroxylon aethiopicum* is used to treat abdominal/colic pain, especially in children. Decoction from the bark of *Osyris lanceolata* is also administered to children to relieve abdominal pains. Ground seeds of *Myrsine africana* are chewed to relive heartburn.

1. **Diarrhoea**

The leaves of *Elaeodendron buchananii* are chewed and swallowed in order to treat diarrhoea. An infusion made from the pounded leaves of *Osyris lanceolata* is used to stop diarrhoea. A decoction of the bark of *Senegalia senegal* is used to treat both diarrhoea and abdominal disorders.

1. **Typhoid**

A decoction made from the boiled fruits of *Commiphora africana* is mixed with milk and administered.

1. **Laxative**

A decoction made from the boiled roots of *Senegalia senegal* has mild purgative effect and is taken to relieve constipation or stomachache.

1. **Emetic**

The pounded roots of *Momordica friesiorum* are mixed with water and administered to induce vomiting.

1. **Anthelminthic agents**

Ground seeds of *Myrsine africana* are used as dewormers. An infusion obtained from the bark of *Olea europaea* *subsp.* *cuspidate* is used to get rid of tapeworms. The bark is pounded, soaked in water and left overnight.

1. **Ulcers**

A preparation made from the pounded leaves of *Dichrostachys cinerea* is used for treatment of ulcers.

1. **Anaesthetic**

An extract from the crushed leaves of *Dichrostachys cinerea* is applied topically to provide local anesthesia.

1. **Respiratory disorders**

An infusion made by soaking the leaves of *Aneilema aequinoctiale* in water is used for the treatment of colds. During an outbreak of common colds, the same infusion is used as a face wash to prevent contracting the colds. The seeds of *Anthriscus sylvestris* are mixed with honey and chewed as a remedy for chesty colds. The roots *Asparagus africanus* are chewed to treat sore throat and cough. Similarly, the roots of *Sida cuneifolia* are chewed and the resultant juice swallowed to relieve sore throats. The juice/sap obtained from either squeezed or chewed roots *Physalis peruviana* is used for treatment of tonsillitis.

A decoction made from the boiled roots of *Carissa edulis* mixed with those of *Olea europaea* *subsp.* *cuspidate, Croton dichogamous* and *Erythrina senegalensis* is used to treat chest pains. Juice obtained from *Solanum incanum* fruits is also taken to treat chest pains. An infusion made from the roots of *Grewia bicolor* is also administered to treat chest pains, while a cupful mixed with cold water is administered for the treatment of colds. A concoction made from the boiled or soaked in water roots and bark of *Toddalia asiatica* is mixed with tea or milk, is also used for the treatment flu and cough. A concoction made from of the roots of *Elaeodendron buchananii* or dried and powdered roots of the same plant is mixed with milk and administered to a patient with haemoptysis (coughing up blood); while berries of *Solanum mauense* are cooked and eaten as cure for pneumonia. A decoction made from either leaves, or roots of *Teclea nobilis* mixed with honey may also be used in the treatment of pneumonia.

1. **Malaria**

The yellow middle bark of *Diospyros abyssinica* is crushed and soaked in water until the solution turns yellow and then sieved before administering to the patient. An infusion prepared by pounding the brown middle bark of *Warburgia ugandensis* and soaking in water may also be used. Milk is added to the infusion and administered to a patient with malaria. A concoction made from the boiled roots *Toddalia asiatica* mixed with milk is also used for the treatment of malaria. An alternative treatment involves the administration of a decoction made from the boiled leaves of *Kigelia africana****.***

1. **Fever**

Pounded roots of *Solanum arundo* are soaked in water and administered or chewed as a remedy for high fever. A decoction obtained by boiling the roots of *Solanum incanum* is also used for the management of fever.

1. **Headache**

A decoction made from the bark of *Kigelia africana* is administered to relive headache.

1. **Toothache**

The fresh top apical leaves of *Dovyalis abyssinica* are chewed and placed around the affected tooth.  *Solanum incanum* roots are rubbed around the affected tooth.

1. **Oral thrush in children**

The flowers of *Acmella calirhiza* are crushed and mixed with water or milk and applied on the affected areas in the child’s mouth.

1. **Venereal diseases**
   1. ***Gonorrhea***

An infusion obtained from mixing the roots of *Asparagus africanus* with water is used to treat venereal diseases, including gonorrhea. An alternative treatment involves the use of a decoction made from the boiled roots of either *Carissa edulis, Croton dichogamous, Dovyalis abyssinica, Erythrina senegalensis*, *Olea europaea* *subsp.* *cuspidate* or *Senegalia senegal* is mixed with sheep tallow. A preparation made from the pounded leaves of *Dichrostachys cinerea* or boiled roots of *Rhamnus prinoides* may also be used. Other treatments include decoction made from the boiled roots of *Osyris lanceolata* mixed milk or meat soup, or concoction made from the boiled roots of *Pavetta subcana* mixed with meat soup or sheep tallow.

- 1. ***Syphilis***

An extract made by soaking the roots of *Acokanthera schimperi* in water is administered to a patient suffering from syphilis, but in a small quantity as the plant is poisonous. The roots of *Elaeodendron buchananii* (a plant known to be poisonous to livestock) are dried, ground to powder and applied on wounds on the affected areas.

- 1. ***Orchitis***

A decoction made from the boiled roots of *Commiphora africana* is used to treat a condition presenting with swollen testicles.

1. **Gynaecological disorders**

The crushed inner bark of *Boscia angustifolia,* mixed with water is used to treat various gynaecological disorders. A decoction made from the boiled roots of *Carissa edulis* mixed with sheep tallow is used to treat lower abdominal pains in pregnancy. A concoction made from the boiled roots of *Sida cuneifolia* mixed with milk is administered to pregnant mothers experiencing excessive foetal activity in order to stabilize the pregnancy.

1. **Abortion**

In order to induce an abortion in pregnant women, a decoction made from the boiled roots of *Ficus sycomorus* is mixed with milk and administered. For pregnant girls, four finger sized pieces of the roots are chewed, which results in abortion after a short while. The roots of *Gloriosa superba* may also be chewed to induce abortion. An infusion obtained from the soaked roots of the same plant may also be administered to achieve the same effect. Alternatively, a decoction made from the boiled roots of *Kigelia africana* may be used.

1. **Ocular disorders**

Juice from *Aneilema aequinoctiale* flowers is applied on the affected eye. An alternative treatment involves the use of a preparation made by pounding the leaves of *Cordia monoica* and soaking in water.

1. **Skin disorders**

Pounded roots of *Albizia gummifera* are mixed with water for bathing to treat skin disorders. Juice from the fruits of *Solanum incanum* may also be applied on the affected areas. A decoction obtained by either boiling or soaking in water the leaves of *Leonotis mollissima* is used to treat skin rashes.

1. **Measles**

A child suffering from measles is washed with an alcoholic beverage made from the fruits of *Kigelia africana*, and then taken to bed and covered heavily to sweat out.

1. **Wounds**

An extract obtained from the leaves of *Asparagus africanus* or *Leonotis mollissima* is used for treating wounds. The leaves are pound and soaked in water. An alternative treatment involves the use of fruit juice of *Solanum incanum.* The fruits are broken and contents applied directly to fresh cuts or wounds. The roots of *Elaeodendron buchananii* may also be used. They are dried, ground to powder and applied on the wounds.

1. **Liver disease**

A decoction made from the boiled leaves of *Olea europaea* *subsp.* *cuspidate* is administered for the treatment of hepatic disorders.

1. **Arthritis**

A concoction made from the boiled roots of *Rhamnus prinoides* or *Achyranthes aspera* mixed with soup is used to treat patients with joint disorders, especially osteoarthritis and related disorders. A concoction made from either leaves, or roots of *Teclea nobilis* mixed with honey may also be used. A decoction made from the boiled bark of *Warburgia ugandensis* or *Zizyphus mucronata* is also used. Alternatively, the dried bark *Warburgia ugandensis* is ground to powder and administered.

1. **Poultice**

Warmed leaves of *Kalanchoe crenata* are used to massage/soothe a painful part of the body.

1. **Mental illness**

An infusion made from the pounded leaves, stem or roots of *Asparagus africanus* and soaked in water is drunk 2 to 3 times a day for treatment of mental illness. A decoction made from the boiled roots of *Cordia monoica* mixed with little milk is used for the same purpose.

1. **Leprosy**

The patient is exposed to steaming/boiling water pot containing the leaves of *Cordia monoica* under a blanket for a period of time. He/she is then washed with the same decoction followed by the application/rubbing of a paste made from the pounded bark of the same plant on his body.

1. **Polio like symptoms**

A decoction made from the roots of *Carissa edulis* mixed with those of *Olea europaea* *subsp.* *cuspidate, Croton dichogamous* and *Erythrina senegalensis* is used to treat a condition which the TMPs described as manifesting with polio like symptoms. We suspected the condition to be a neuromuscular system disorder.

1. **Anthrax**

A decoction made from the roots of *Solanum mauense* is used to treat anthrax in both humans and animals.

1. **Malnutrition**

An infusion made from the leaves of *Aneilema aequinoctiale* is administered to children as a remedy for malnutrition.

1. **Appetizer**

A decoction from boiled roots *Clutia abyssinica* is mixed with soup and administered in order to improve appetite. An infusion made from pounded leaves of *Prunus africana* soaked in water may also be used.

1. **Blood purifier**

A decoction obtained by either boiling or soaking in water the leaves of *Leonotis mollissima* is used to cleanse the blood.

1. **Snake bites**

An extract obtained by chewing the bark of *Commiphora africana* combined with tobacco is applied on the bitten area. An alternative treatment involves the chewing of the tender leaves of *Solanum incanum* and rubbing the extract including the leaves hard into the bitten area. An infusion made by soaking the roots of *Grewia bicolor* or *Zizyphus mucronata* in water is also administered for snake bite treatment.

1. **Miscellaneous uses**
   1. ***Preservative***

A decoction made form pounded stem of *Rhamnus prinoides* mixed with water is used as a preservative.

- 1. ***Arrow poison***

An infusion from the bark of *Acokanthera schimperi* soaked in water is used as an arrow poison.

1. **Veterinary uses**
   1. ***East Coast Fever***

The flowers of *Aerva javanica* are ground into paste, mixed with water and administered to cattle to treat East Coast Fever. An alternative treatment involves use of *Boscia angustifolia* leaves which are pounded and soaked in water.

- 1. ***Throat cancer in cattle***

The whole of *Galium aparinoides* herb is pounded and soaked in water or boiled and decoction administered.

***34.3 Respiratory disorders***

An extract obtained by soaking the leaves and stem of *Cissus fischeri* is administered to cattle with respiratory disorders, whereas juice from *Solanum incanum* fruits is squeezed into the sheep’s nostrils to treat cough. An infusion made by soaking the whole of *Pavetta subcana* plant is administered to calves to relieve cough. It is also used to wash them to get rid of fleas.

- 1. ***Ocular disorders***

The yellow middle bark of *Diospyros abyssinica* is crushed and soaked in water until the solution turns yellow and then sieved. The drops are then applied onto the affected eye.
